# Gastropexy may reduce symptomatic recurrence in paraesophageal hernia repair: a Scandinavian retrospective multicenter study

**DOI:** 10.1093/dote/doag051

**Published:** 2026-05-21

**Authors:** Lene Osterballe, Magnus H Fasting, Eirik K Aahlin, Rasmus Goll, Tom Wilsgaard, Torgeir T Søvik, Mahdi Alamili, Cecilie B Lassen, Palle B Miliam, Per-Even Storli, Mads Vikhammer Gran, Kim E Mortensen

**Affiliations:** Department of Gastrointestinal Surgery, University Hospital of North Norway, Harstad, Norway; Institute of Clinical Medicine, UiT the Arctic University of Norway, Tromsoe, Norway; Department of Gastrointestinal Surgery, Oslo University Hospital Ullevaal, Oslo, Norway; Institute of Clinical Medicine, UiT the Arctic University of Norway, Tromsoe, Norway; Department of Gastrointestinal Surgery, University Hospital of North Norway, Tromsoe, Norway; Institute of Clinical Medicine, UiT the Arctic University of Norway, Tromsoe, Norway; Department of Gastroenterology, Division of Internal Medicine, University Hospital of North Norway, Tromsoe, Norway; Department of Community Medicine, UiT, Arctic University of Norway, Tromsoe, Norway; Department of Gastrointestinal Surgery, Oslo University Hospital Ullevaal, Oslo, Norway; Department of Gastrointestinal Surgery, Copenhagen University Hospital - Hvidovre, Copenhagen, Denmark; Department of Gastrointestinal Surgery, Copenhagen University Hospital - Hvidovre, Copenhagen, Denmark; Department of Gastrointestinal Surgery, Copenhagen University Hospital - Hvidovre, Copenhagen, Denmark; Department of Gastrointestinal Surgery, St. Olavs Hospital, Trondheim University Hospital, Trondheim, Norway; MiDT, National Research Center for Minimally Invasive and Image-Guided Diagnostics and Treatment, St. Olavs Hospital, Trondheim University Hospital, Trondheim, Norway; MiDT, National Research Center for Minimally Invasive and Image-Guided Diagnostics and Treatment, St. Olavs Hospital, Trondheim University Hospital, Trondheim, Norway; Institute of Clinical Medicine, UiT the Arctic University of Norway, Tromsoe, Norway; Department of Gastrointestinal Surgery, University Hospital of North Norway, Tromsoe, Norway

**Keywords:** anterior gastropexy, anti-reflux surgery, foregut surgery, large hiatal hernia, Nissen fundoplication, paraesophageal hernia

## Abstract

Paraesophageal hernia (PEH) repair is challenging, with high recurrence rates reported. Evidence suggests that anterior gastropexy may reduce the recurrence rate, but real-world data are lacking. We performed a retrospective multicenter study of adults undergoing PEH repair (2018–2023) at four Scandinavian centers. The primary outcome was a computed tomograpy (CT) verified recurrence defined radiologically as a ≥20 mm herniation above the diaphragm. Propensity score matching balanced patients with and without anterior gastropexy. Outcomes and predictors were analyzed using matched comparisons and Cox proportional hazards regression. A total of 395 patients underwent primary PEH repair during the study period. The median age was 70 (range 63–76) years, and the median follow-up was 1.8 (0.8–3.3) years. 297 (75%) patients received a gastropexy as part of the procedure, while 98 (25%) patients did not. The group without gastropexy had a significantly higher rate of recurrence, 33 out of 98 patients (34%) compared with the gastropexy group, 45 out of 297 patients (15%, *P* < 0.001). After propensity score matching and adjustment for confounding factors, the risk of recurrence remained higher for patients who did not receive gastropexy (HR: 2.5, 95% CI: 1.3–4.9; *P* = 0.007). Anterior gastropexy was associated with a significantly lower risk of recurrence following PEH repair. These findings indicate that this component of the surgical procedure might improve long-term outcomes.

## INTRODUCTION

The optimal surgical approach for paraesophageal hernia (PEH) (grades II−IV hiatal hernia) repair remains a subject of ongoing debate.^1,2^ High recurrence rates up to 57 % are reported regardless of the use of mesh reinforcement.^3,4^ For many years, PEH repair with gastropexy in various forms has been employed to reduce recurrence rates, though clear evidence supporting its protective effect is lacking. In 1955, Boerema first described the anterior gastropexy technique.^5^ However, this method was largely abandoned for several decades. In recent years, it has reemerged due to favorable outcomes reported in retrospective studies,^6–9^ prospective studies^10^ and recently published reviews.^11,12^ Nevertheless, it is not included in the latest SAGES guidelines for hiatal hernia repair,^1^ unlike the earlier version.^13^ In the 2023 EAES guidelines, fundoplication is recommended over gastropexy for elective PEH repair whereas gastropexy is favored over fundoplication in frail, elderly patients and in acute operations.^2^ In 2024, the first randomized controlled trial on gastropexy in PEH repair demonstrated that incorporating an anterior gastropexy significantly reduces recurrence rates.^14^ These findings support gastropexy as a promising strategy to address the persistent issue of recurrence in PEH repair. The procedure is considered low-risk, quick, and easy to perform, making it a potential attractive adjunct to PEH repair.

In light of growing evidence suggesting that PEH repair with gastropexy decreases CT-verified recurrence, we aimed to investigate this through a Scandinavian multicenter study using real-world data.

## METHODS

### Study population

This study is a retrospective study of patients undergoing PEH repair at four surgical centers in Scandinavia (University Hospital of North Norway, St. Olav University Hospital, Copenhagen University Hospital - Hvidovre and Oslo University Hospital) from November 2018 to November 2023. Parts of the study population have been analyzed for different outcomes previously.^15^

All patients over 18 years of age who were operated for PEH in an elective setting without the use of Roux-en-Y-reconstruction were included. Patients with previous hiatal or bariatric surgery were excluded. Indications for surgery were relevant symptoms combined with a CT scan and endoscopy showing a PEH with more than 30% of the stomach including the fundus above the diaphragmatic hiatus.

Patients were routinely followed up within 12 months after PEH repair in the outpatient clinic, either in person or by telephone. The perioperative routines at the participating sites are described in Table 1. CT scans were performed, primarily based on the presence of ongoing or new symptoms at follow-up (Table 1). One center (Oslo University Hospital, OUS) partly performed routine CT scans at 12 months postoperatively (Table 1), therefore a sensitivity analysis was conducted using a Cox proportional hazard analysis, excluding patients from OUS from the raw data when calculating the risk of recurrence. The end of the follow-up period was defined as the time to death, recurrence or study end in February 2024, with a median follow-up time calculated accordingly. All participating sites have regional responsibility for PEH repair, with systematic re-referral in case of recurrence.

**Table 1:** Per- and postoperative routines at each participating site during the study period from November 2018 to November 2023

		Peroperative procedure steps			Postoperative follow-up			
Centre	Number of included patients (total N = 395)	Gastropexy	Fundoplication	Mesh	Routine follow-up time after primary PEH repair	Time under observation (years)*	Follow up CT scan	Recurrence on CT
Hvidovre Hospital	243 (61)	226 (93)	18 (7)	32 (8)	at 3 months	2.4 (0.9–3.6)	if indicated	40 (16)
Oslo University Hospital	71 (18)	0	59 (83)	49 (69)	at 12 months	1.7 (0.8–3.8)	routine at 12 months from 11/2018–12/2021 and from 01/2022–11/2023 if indicated	21 (30)
St Olav University Hospital	66 (17)	59 (89)	63 (95)	14 (21)	at 12 months	1.4 (0.6–2.5)	if indicated	16 (24)
University Hospital of North Norway	15 (4)	12 (80)	3 (20)	0	at 3 months	1.4 (1.4–1.7)	If indicated	1 (7)

^*^Time under observation is defined as the time from PEH procedure to time to recurrence, end of study period or death. Values are median (inter-quartile range). Some patients underwent both gastropexy and fundoplication. Hence, the number of procedure steps does not necessarily match the total number of procedures at each hospital.

Values are numbers and percentage in parentheses unless indicated otherwise.

### Study variables

The main study outcome was the presence of a CT-verified recurrence of a hiatal hernia. Recurrence on CT was defined as a gastric or any other organ herniating 20 mm or more above the level of the diaphragmatic crus.

Exposure variables included patient age, patient sex, height (cm), weight (kg), BMI (kg/m^2^), patient comorbidity using the Charlson Comorbidity Index,^16^ use of mesh, anti-reflux procedure, and postoperative complications defined as a Clavien-Dindo grade of ≥3.^17^

### Surgical technique

The gastropexy was performed as an anterior gastropexy, anchoring the stomach to the anterior abdominal wall. This was achieved laparoscopically using a continuous nonabsorbable suture or transcutaneously with three interrupted nonabsorbable sutures. Otherwise, the surgical procedures were the same with the removal of the hernia sac and abdominal contents from the mediastinum, ensuring adequate esophageal length and hiatal closure with non-absorbable sutures. The Collis procedure was not performed routinely at any of the centers. A fundoplication was performed at the surgeon’s discretion, using a 360-degree ‘Nissen’ or 270-degree ‘Toupet’ technique. A hiatal pre-shaped biological mesh was also used at surgeon’s discretion.

### Ethics

The study was conducted in accordance with the Declaration of Helsinki.^18^ The use of data was in agreement with the Data Protection Officers at the participating institutions in Denmark (F-23057940) and Norway (ref. 03222, 662003, and 21-13019, respectively).

### Statistical analysis

We performed a propensity score matching between the patients receiving an anterior gastropexy and those who did not to reduce potential confounding factors and balance baseline characteristics between the groups. Propensity scores were calculated using a logistic regression model, with covariates among potential predictors of outcome. We analyzed our dataset to identify associations between the independent variables and the dependent variable, which formed the basis for the variables included in the propensity score. The dependent factor was gastropexy and independent covariates included age, sex, body mass index, comorbidity, grade of PEH, perioperative mesh use and fundoplication. A 1: 1 and 1:2 nearest-neighbor matching algorithm was applied, pairing each gastropexy-treated subject to the subject with the closest propensity score who did not get gastropexy. Balance between groups was assessed using standardized mean differences, with a threshold of <0.05. The propensity score match was performed in SAS. Statistical analysis was performed in SPSS. Continuous variables were presented as median (interquartile range). For Gaussian distributed data, parametric tests were used (independent samples student’s *t-*test, two-sided) while non-Gaussian distributed data were tested by a non-parametric method (Mann–Whitney U). Categorical variables were reported as numbers with percentages and compared using the chi-squared or Fisher’s exact test as appropriate. Cox proportional hazards regression analysis was utilized for factors influencing recurrence. We checked the assumption of proportional hazards by examining log–log plots in the final model and found no violations. Median follow-up was calculated using the reverse Kaplan–Meier. A *P*-value < 0.05 was considered statistically significant.

## RESULTS

A total of 395 patients were included in the study: 243 patients from Copenhagen University Hospital - Hvidovre, 15 patients from the University Hospital of North Norway, 66 patients from St. Olav University Hospital and 71 patients from Oslo University Hospital. The gender distribution was 25% male (100) and 75% female (295). The median age was 70 (range 63–76) years. The median follow-up was 1.8 (0.8–3.3) years. Propensity score matching of the 98 patients without anterior gastropexy to the 297 patients with gastropexy, resulted in two matched groups of 66 and 91 patients, respectively. After propensity score matching, the groups were comparable, with similar baseline characteristics, peri- and post-operative characteristics (Table 2). CT-verified recurrence was higher in the group without gastropexy both before (33 (34%) vs. 45 (15%), *P* < 0.001) and after propensity score matching (23 (35%) vs. 16 (18%), *P* = 0.011).

**Table 2:** Patient demographics on PEH repair with or without gastropexy. Before and after propensity score match

Variable	Before propensity score match			After propensity score match		
	PEH repair without gastropexy (*N* = 98)	PEH repair with gastropexy (*N* = 297)	*P*-value^†^	PEH repair without gastropexy (*N* = 66)	PEH repair with gastropexy (*N* = 91)	*P*-value^†^
Sex (F)	65 (66)	230 (77)	0.028	49 (74)	66 (73)	0.857
Age/years	68 (60–71)^*^	71 (65–78)^*^	0.002^‡^	69 (61–74)^*^	69 (63–76)^*^	0.762^‡^
BMI	27 (25–30)^*^	27 (25–30)^*^	0.625^‡^	27 (25–30)^*^	27 (25–30)^*^	0.981^‡^
CCS ≥ 3	29 (30)	130 (44)	0.008	22 (33)	29 (32)	0.979
Grade of PEH						
III	84 (86)	254 (86)	0.963	54 (82)	80 (88)	0.361
IV	14 (14)	43 (14)		12 (18)	11 (12)	
Fundoplication	82 (84)	61 (21)	<0.001	51 (77)	61 (67)	0.211
Mesh use	51 (50)	44 (15)	<0.001	27 (41)	28 (31)	0.236
Postoperative events (CD-score ≥ 3)	5 (5)	18 (6)	0.725	5 (8)	6 (7)	1.000
CT-verified recurrence	33 (34)	45 (15)	<0.001	23 (35)	16 (18)	0.011

^*^Values are median (IQR).

^†^ *x*  ^2^-test or Fischer’s exact test as appropriate.

^‡^Independent samples *t-*test. CCI, Charlson Comorbididty index; CD, Clavien-Dindo; PEH, paraesophageal hernia.

Categorical variables have values in percentage in parentheses unless indicated otherwise.

**Fig. 1:**
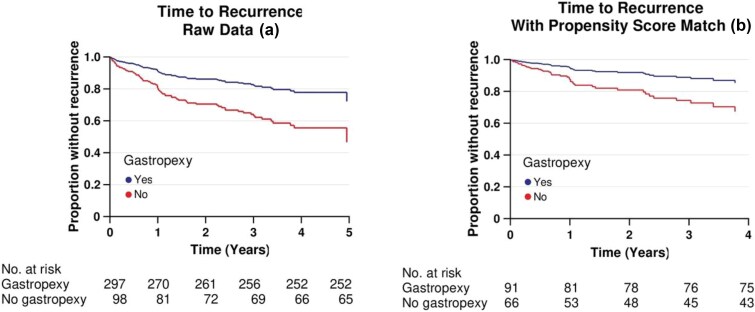
Anterior gastropexy and time to recurrence. Cox proportional hazards regression analysis evaluating gastropexy as a protective factor against recurrence. Fundoplication, mesh use, and gastropexy were included in the multivariate regression model, with gastropexy identified as the only significant risk factor for recurrence. (a) Before propensity score matching (HR: 2.3; CI 1.3–4.4; p = 0.008), and (b) after propensity score matching (HR: 2.5; CI: 1.3–4.9; p = 0.007).

Figure 1 illustrates the time to recurrence before and after propensity score matching. In both cases, the data analysis shows a significantly higher risk of recurrence in patients who did not undergo anterior gastropexy (HR: 2.3; CI: 1.3–4.4; *P* = 0.008 and HR: 2.5; CI: 1.2–4.9; *P* = 0.007, respectively). The regression analysis was adjusted for mesh use and fundoplication.

The sensitivity analysis (excluding patients from OUS) showed a similar decreased risk of recurrence associated with gastropexy (HR: 0.3; CI: 0.1–0.5; *P* < 0.001).

## DISCUSSION

Our real-world data from four Scandinavian centers on PEH repair show that the use of anterior gastropexy was associated with a lower risk of CT-verified recurrence, both before and after propensity score matching. This finding aligns with a recent study by Petro *et al*.^14^ However, our study adds data from a different population. Despite the well-known limitations of a retrospective design, it is noteworthy that a simple procedure such as gastropexy might significantly impact recurrence rates.

The potential side effects of gastropexy, including pain, are important considerations. Evidence from the above-mentioned study^14^ indicates that gastropexy may cause pain and, in some cases, necessitate reoperation. Although we did not observe any reoperations due to severe discomfort from the gastropexy in our study, it is a subject of importance for future studies.

Several limitations of this study must be acknowledged. One limitation is the difference in CT scan indications at different institutions (Table 1). This heterogeneous practice of CT follow-up represents a possible bias. However, in the sensitivity analyses excluding the institution with the differing follow-up, the reduced risk associated with gastropexy persisted.

Other limitations are the retrospective study design and unsystematic follow-up after the first postoperative year. A longer active follow-up period could have provided more comprehensive insights into recurrence rates, as demonstrated in another retrospective study with a median follow-up of 22 years, which reported a treatment failure rate of 36.7% for PEH repair.^19^

We observed that older patients with higher Charlson Comorbidity Scores (CCS) were more frequently represented in the gastropexy group. This likely reflects a clinically tailored approach in practice, where patient characteristics influence the choice of surgical technique, as recommended by the EAES guidelines.^2^ This introduces a potential confounding factor that must be considered. That said, we have addressed this by including CCS in the propensity score analysis.

We did not record the specific reasons for the use of fundoplication and/or mesh at the participating centers. This may be due to the traditions at the different centers, but it could also be related to larger defects where mesh and/or fundoplication were deemed necessary. To address this, we adjusted for these factors in our propensity score by including mesh use and fundoplication, which led to a propensity score matching process that necessitated the exclusion of many patients, resulting in stricter criteria, smaller groups, and consequently reduced reliability. This was primarily due to the heterogeneity of the groups at baseline characteristics and the inclusion of the perioperative variables of mesh use and fundoplication, which were associated with the dependent factor (gastropexy). However, both the regression analysis performed on all patients and the propensity score analysis performed on a subgroup of patients showed similar results. Nevertheless, the surgical heterogeneity observed among the involved hospitals (Table 1) represents a clear limitation of this study.

Patient-reported outcome measurements were not available in our study and should be a focus of future prospective trials.

In conclusion, this study supports the use of anterior gastropexy in PEH repair, suggesting its potential to lower recurrence rates. Despite its limitations, the strength of our study lies in being, to our knowledge, the first large-scale European multicenter study, presenting real-world data on gastropexy in PEH repair.

## Notes

Specific author contributions: Conception and design of study, statistical analysis, writing original draft and writing—review and editing: Lene Osterballe, Magnus Fasting, Eirik Kjus Aahlin; Conception and design of study, statistical analysis, writing original draft and writing—review and editing: Torgeir T. Soevik; Conception and design of study, statistical analysis and writing—review and editing: Rasmus Goll; Statistics: Tom Wilsgaard; Conception and design of study, data collection, writing-review and editing: Mahdi Alamili; Conception and design of study, data collection, writing—review and editing: Per-Even Storli; Data collection, writing—review and editing: Mads Vikhammer Gran; Data collection and writing—review and editing: Cecilie Brandt Lassen; Design of study, writing—review and editing: Palle Beck Miliam; Conception and design of study, statistical analysis, writing original draft and writing—review and editing: Kim Erlend Mortensen.
